# Standardisation in robotic surgery for inflammatory bowel disease: a systematic review

**DOI:** 10.1007/s11701-026-03194-y

**Published:** 2026-02-21

**Authors:** Ayesha Unadkat, Shobhit Arya, Aliki Rompou, Valerio Celentano

**Affiliations:** 1 Department of Surgery, Imperial College London, Exhibition Road, London, SW7 2AZ England; 2 Department of Colorectal Surgery, Chelsea and Westminster Hospital, 369 Fulham Rd., London, SW10 9NH England

**Keywords:** Robotic surgery, Inflammatory bowel disease, Crohn’s disease, Ulcerative colitis, Surgical standardisation, Systematic review

## Abstract

Robotic-assisted surgery offers technical advantages over laparoscopy, including improved dexterity and visualisation. However, its role in inflammatory bowel disease (IBD) remains poorly defined, with existing studies limited by variability and lack of standardisation. This systematic review aimed to evaluate the reproducibility, operative detail, outcome reporting, and procedural consistency in the current literature on robotic-assisted surgery for IBD. A systematic review was conducted following PRISMA 2020 guidelines and registered on PROSPERO (CRD42024514488). Comprehensive searches of five databases and grey literature from January 2015 to April 2024 were performed. Studies involving robotic surgery in adult IBD patients were included. Methodological quality was assessed using the Newcastle–Ottawa Scale. Sixteen studies involving 614 patients met inclusion criteria. Most were retrospective (81.3%) and single-arm (62.5%), with robotic ileocolic resection being the most common procedure (50%). Significant heterogeneity existed in port placement, docking, and intraoperative techniques. Technical reporting, particularly on robotic setup, was inconsistent. Definitions of postoperative outcomes, including complications and conversion rates, varied across studies. Enhanced Recovery After Surgery protocols were used in 18.8% of studies, with minimal reporting of patient-reported outcomes. While risk of bias was generally low, limited follow-up and absence of comparator arms reduced the strength of conclusions. Current evidence on robotic surgery in IBD is methodologically variable and poorly standardised, particularly regarding technical setup and outcome definitions. Future research should focus on prospective, multicentre studies with detailed intraoperative data, standardised outcomes, and long-term follow-up.

## Introduction

Despite significant advances in medical therapy, surgical intervention remains a cornerstone in the management of inflammatory bowel disease (IBD) with up to 80% of patients with colonic or perianal Crohn’s disease (CD) requiring surgery during their lifetime [1]. The operative management of CD presents unique and persistent challenges due to the recurrent, transmural, and often multifocal nature of the disease, which leads to complex surgical fields marked by dense adhesions, active inflammation, and the consequences of prior resections. Traditionally, open surgery has been the default approach in these scenarios due to such complexity. However, this approach is associated with significant drawbacks, including higher perioperative morbidity, increased risk of surgical site infections, prolonged convalescence, and a negative biopsychosocial impact. Minimally invasive surgery (MIS), particularly laparoscopic techniques, has demonstrated meaningful clinical benefits in colorectal procedures more broadly, including reduced postoperative pain, shorter hospital stays, and improved cosmetic outcomes [2]. 

Nonetheless, the application of MIS in IBD has been variable and frequently limited. This inconsistency is largely attributable to the technical demands of operating in a chronically inflamed, anatomically distorted field, often complicated by dense adhesions, fistulas, mesenteric foreshortening, and fibrotic tissue planes [3]. 

Robotic-assisted surgery, which offers enhanced dexterity, superior three-dimensional visualisation, tremor filtration, and increased degrees of instrument articulation, represents an intuitively advantageous platform for addressing the unique anatomical and pathological challenges encountered in CD. These features may offer distinct advantages in complex reoperative or anatomically confined fields. Despite this, the uptake of robotic platforms in IBD remains modest and inconsistent [4].

While robotic systems have gained widespread traction in oncological colorectal surgery, their use in IBD—despite their theoretical benefits—has lagged considerably. The disparity in adoption between malignant and benign colorectal pathology suggests a lack of robust evidence specific to the inflammatory pathology of IBD. Therefore, we aim to systematically review the current evidence base regarding robotic-assisted surgery for IBD with a focus on the consistency of outcome reporting, technical detail, and degree of procedural standardisation in the existing literature.

## Methods

This systematic review was reported in accordance with the Preferred Reporting Items for Systematic Reviews and Meta-Analyses (PRISMA) 2020 guidelines [5]. To ensure methodological transparency and facilitate reproducibility, the review protocol was prospectively registered with the International Prospective Register of Systematic Reviews (PROSPERO) under the registration number CRD42024514488 [6]. 

A comprehensive literature search was performed across five major electronic databases: Cochrane Library, MEDLINE, PubMed, Web of Science, and Google Scholar, covering the period from January 2015 to the present. This timeframe was chosen to reflect the global adoption of 4th generation robotic platforms, capturing modern IBD practices rather than just the early, cancer-focused results of early adopters [7]. No restrictions were applied with respect to language, publication status, or article type, in order to maximise search sensitivity and minimise selection bias. The inclusion of studies from 2015 onwards was intended to reflect contemporary surgical practice following widespread adoption of robotic platforms in colorectal surgery.

In addition to peer-reviewed databases, grey literature sources were systematically searched to capture the most recent data in this rapidly evolving field. This included clinical trial registries (ClinicalTrials.gov, World Health Organization International Clinical Trials Registry Platform, and ISRCTN registry), relevant conference proceedings from the European Society of Coloproctology, European Association for Endoscopic Surgery, Society of American Gastrointestinal and Endoscopic Surgeons, and American Society of Colon and Rectal Surgeons from 2022 onwards, as well as conference abstracts, National Health Service policy reports, dissertations, and theses.

The search strategy employed a combination of Medical Subject Headings (MeSH), free-text terms, and Boolean operators (‘AND’, ‘OR’). Core search terms included: *“inflammatory bowel disease*,*” “Crohn’s disease*,*” “ulcerative colitis*,*” “pouch*,*” “robotic surgery*,*” “port placement*,*”* and *“trocar”*. Reference lists of included studies and relevant review articles were manually screened for additional eligible publications.

Study screening and selection were conducted using Covidence software (Veritas Health Innovation, Melbourne, Australia), with automatic deduplication of records. Detailed eligibility criteria are summarised in Table 1. Two independent reviewers (AU and SA) performed title and abstract screening, followed by full-text review of potentially eligible studies. Disagreements regarding study inclusion or data extraction were resolved through discussion with an independent third reviewer (AR) and subsequent group consensus where required.

**Table 1:**
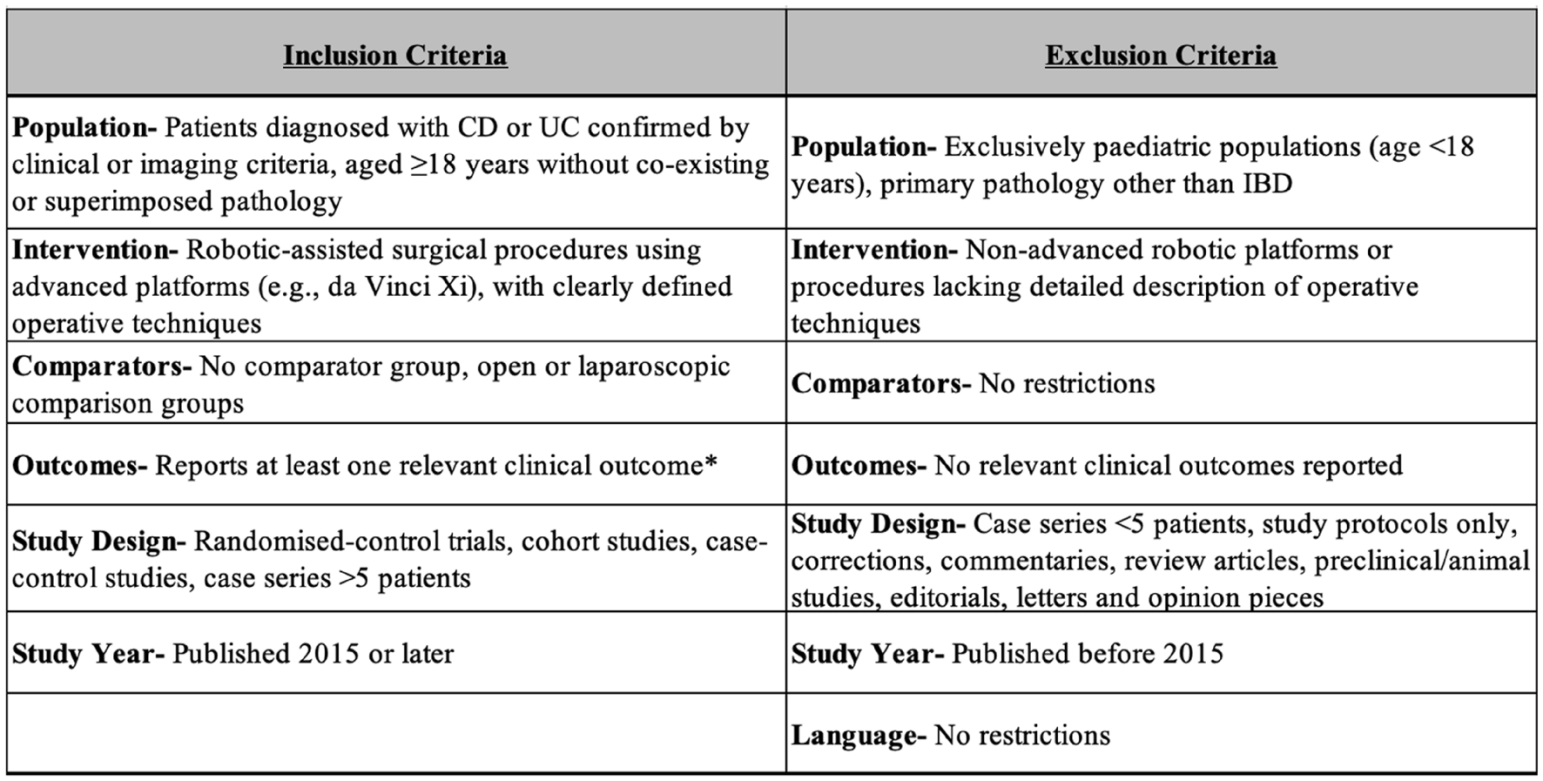
Inclusion and exclusion criteria for systematic review studies on robotic-assisted surgical procedures in IBD patients. Criteria are categorised by population, intervention, comparator, outcomes, study design, and publication year *Clinically relevant outcomes included but were not limited to conversion to open surgery, complication rates, length of hospital stay, stoma formation, reoperation rates, mortality rates, anastomotic leak, functional outcomes (e.g. time to stool). CD indicates Crohn’s Disease; UC, ulcerative colitis; IBD, inflammatory bowel disease

**Fig. 1:**
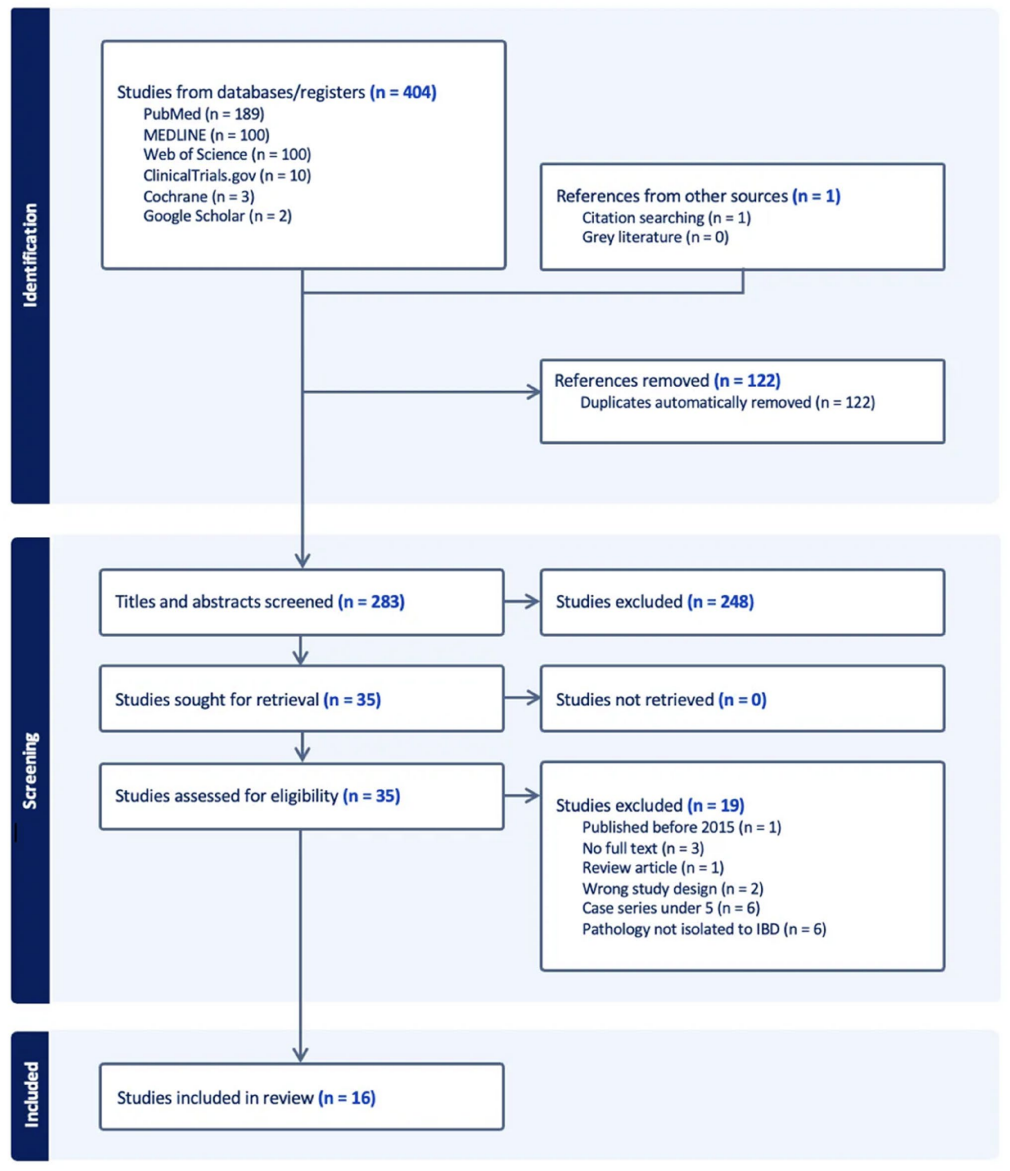
PRISMA 2020 flow diagram illustrating the study selection process for inclusion in the systematic review on robotic surgery for inflammatory bowel disease. A total of 405 records were initially identified; following de-duplication and screening for eligibility, 16 studies met the inclusion criteria and were subsequently included in the final review. IBD indicates inflammatory bowel disease

A PRISMA-compliant flow diagram summarising the study selection process is presented in Fig. 1.

For data extraction, a customised extraction form was developed in Microsoft Excel, informed by the methodology outlined in the Cochrane Handbook for Systematic Reviews of Interventions [8]. The form was piloted on a subset of included studies to ensure clarity, consistency, and relevance, and refined accordingly prior to full data collection.

The data extraction framework captured variables across four core domains:

1. Study characteristics – including author publication history, year of publication, country of origin, and study design;
2. Patient demographics – including age, sex, BMI, and prior surgical history;
3. Surgical technique – with emphasis on operative approach, robotic platform used, and port placement strategy;
4. Primary clinical outcomes – including intraoperative conversion rates, postoperative complication rates, and markers of functional recovery.

Extracted data were tabulated and subjected to descriptive synthesis. Quantitative data were analysed using Microsoft Excel. Continuous variables were summarised with mean (standard deviation [SD]) and median (interquartile range [IQR]) for parametric and non-parametric data, respectively, while categorical data were presented as frequencies and percentages.

All included studies were observational in design. Methodological quality was assessed using the Newcastle–Ottawa Scale (NOS) [9], which applies a star-based scoring system across three domains: selection, comparability, and outcome assessment. Studies were classified as:

- Low risk of bias (score = 9),
- Moderate risk (score = 7–8),
- High risk (score < 6).

Where appropriate, a one-point penalty was applied for major methodological limitations (e.g., lack of control group or incomplete outcome data), while minor concerns (e.g., limited baseline matching) warranted a half-point deduction. Full scoring criteria and rationales are provided in Appendix 1.

## Results

### Study characteristics and patient demographics

The initial search yielded 404 records, of which 16 studies met the inclusion criteria following de-duplication and screening (Fig. 1). The majority of studies were retrospective in design (81.3%, *n* = 13) [10–20, 23, 25] and conducted in the United States (56.3%, *n* = 9). [10–16, 21, 22] First authors predominantly (93.8%, *n* = 15) had established publication history in robotic surgery [10–16, 21, 22, 24, 25]. Sample sizes within robotic cohorts ranged from 6 to 109 patients (*median* = 31; *IQR* = 46).

Of the included 16 studies, CD was listed as an indication in 12 papers [10, 12–18, 22–25], UC in 8 papers [13, 16, 19–21, 24, 25]. Four studies [16, 21, 24, 25] included mixed IBD cohorts, where both CD and UC were listed as indications. Robotic ileocolic resection (robotic ICR) was the most performed procedure (50%, *n* = 8) [10–15]. Comparator arms included laparoscopic surgery (31.3%, *n* = 5) [16, 19, 20, 22, 23], open surgery (6.3%, *n* = 1) [10], while 62.5% (*n* = 10) were single-arm studies [11–15, 17, 18, 21, 24, 25]. All studies reported age and sex. Pooled mean patient age was 41.15 years (SD = 7.82), mean body mass index (BMI) was 23.62 kg/m² (*SD* = 2.07; reported in 14 studies) [10–13, 15–21, 24, 25], and males constituted 49.5% (*SD* = 19.5%) of cohorts. Prior abdominal surgery was reported in half (*n* = 8)^11–16,19,20^, though details were inconsistently documented. Only one study (6.3%) reported on surgeon training, noting that cases were performed during the surgeons’ robotic learning curve [20]. Risk of bias assessment indicated 14 studies at low risk and 2 at moderate risk, common limitations included short follow-up (≤ 30 days) and inadequate baseline matching.

### Surgical setup

The da Vinci platform was predominantly used (93.75%); one employed the Medtronic Hugo system (6.25%) [25]. Preoperative planning was described in five studies (31.3%) [16, 20, 21, 23, 24], with platform selection driven primarily by robotic availability in 12.5% (*n* = 2) [16, 20]. Preoperative imaging guided surgical planning in three studies (18.8%) [21, 23, 24].

For colectomy and ICR, patient positioning was reported in 25% (*n* = 4), [11, 21, 23, 24] the most common being modified lithotomy (*n* = 2) [19, 23], left lateral decubitus (*n* = 1) [21], and Trendelenburg (*n* = 1) [11]. Robotic docking strategies were specified in 18.8% (*n* = 3) [22–24] all utilising left-sided approaches. Double-docking was employed in 12.5% (*n* = 2) [23, 24]; the remainder used single-docking techniques. Port placements were comprehensively detailed in 31.3% (*n* = 5) [17, 20, 21, 23, 24], with no consistent approach identified.

### Intraoperative techniques

Technical details were inconsistently reported and varied widely: dissection of target anatomy [11, 14, 16–18, 21, 25] and bowel mobilisation [15, 17–19, 21, 23, 25] were described in 43.8% (*n* = 7) each and vascular control in 56.3%, (*n* = 9) [11, 15–21, 25]. No common operative approach emerged. Extraction site was reported in 50% (*n* = 8) [11, 15–18, 21, 23, 24], most frequently as Pfannenstiel incisions (25%, *n* = 4) [11, 15, 18, 24].

Anastomotic technique was described in 75% (*n* = 12) [11, 15–21, 25, 26]; one study applied a BMI-based approach [21]. Side-to-side anastomosis was preferred (*n* = 4) [11, 15–17], including one series using fully intracorporeal anastomosis (ICA) [15] and another comparing intra- versus extracorporeal anastomosis (ECA) [11]; alongside stapled J-pouch formation (*n* = 3) [16–18], and the Kono-S technique (*n* = 1) [25].

### Postoperative outcomes

Enhanced Recovery After Surgery (ERAS) protocols were reported in 18.8% (*n* = 3) [11, 15, 16]. Postoperative outcomes were variably reported and are summarised in Table 2.

**Table 2:**
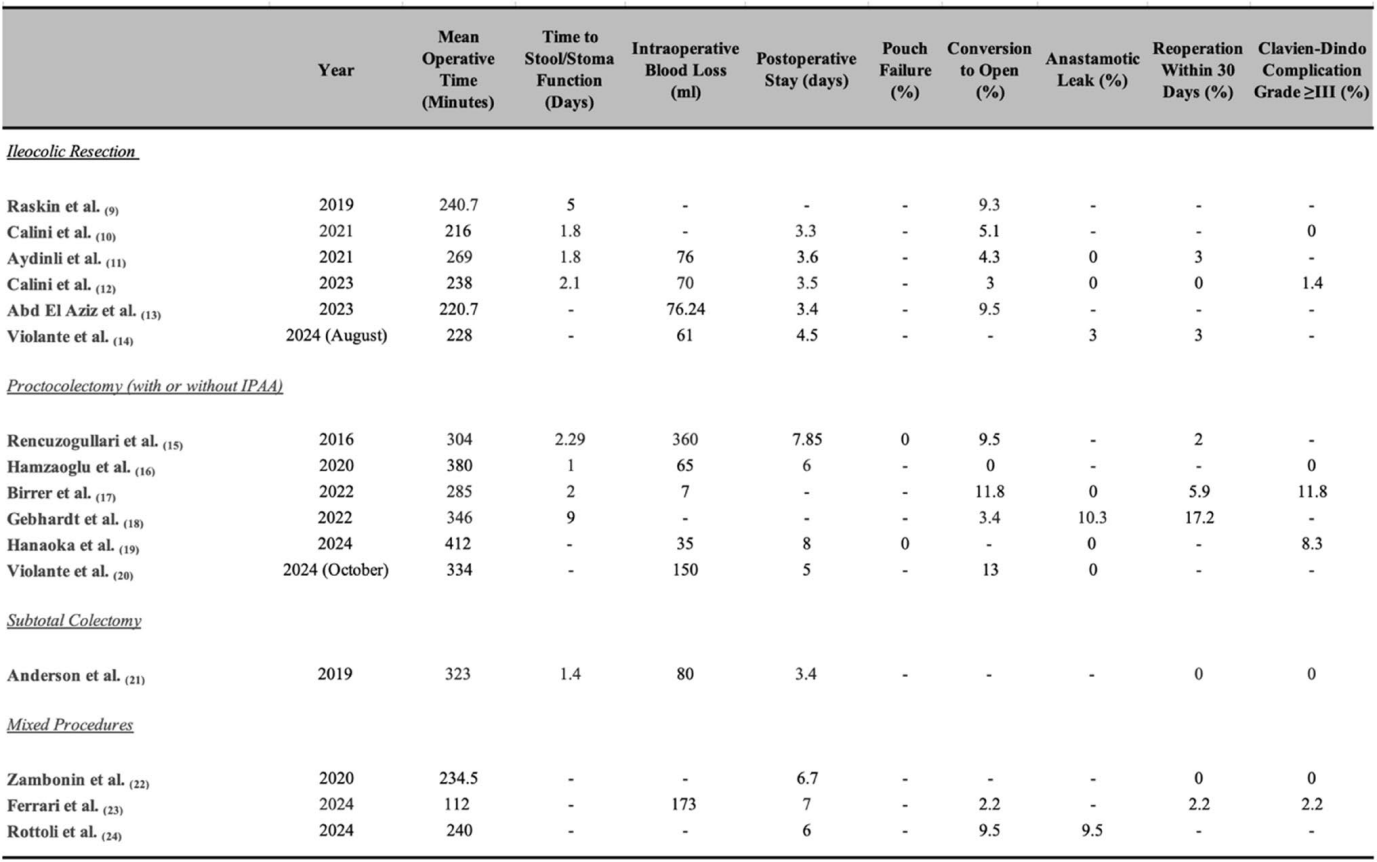
Summary of postoperative outcomes following robotic inflammatory bowel disease surgery across studies. The extent and nature of postoperative outcome reporting varies between studies. Reported variables include mean operative time (minutes), time to return of bowel function (stool or first flatus; days), intraoperative blood loss (mL), postoperative length of stay (days), pouch failure rate (%), conversion to open surgery (%), anastomotic leak rate (%), 30-day reoperation rate (%), and Clavien-Dindo grade ≥III complication rate (%). Only outcomes reported in more than one study were considered

A dash (–) denotes that the outcome was not reported in the corresponding study

### Ulcerative colitis subgroup analysis

Of the eight studies (50%) including patients with ulcerative colitis (UC), the most frequently performed procedures were proctocolectomies with or without ileal pouch anal anastomosis (IPAA), either as single-stage or staged approaches [16–21]. Robotic-assisted proctocolectomy was associated with acceptable perioperative outcomes, including low conversion rates (range: 0–13%) and comparable complication profiles to laparoscopic and open approaches [16–19, 21]. Reported postoperative morbidity included anastomotic leaks (0–10.3.3%) [18–21], pouch failure (*MEAN* = 0%) [16, 20], and 30-day reoperation rates (0–17.2.2%) [16, 18, 19]. Functional outcomes, specifically, time to stool/stoma function was reported in two studies, with no significant difference between robotic and laparoscopic cohorts [16, 17]. Descriptions of perioperative care and use of ERAS protocols were seen in 0% of UC studies, with compliance and individual component adherence were unclear. Long-term outcome data and patient-reported measures were limited or absent across studies.

## Discussion

This systematic review identified persistent limitations across the current body of literature on robotic surgery for IBD. Most included studies were retrospective in design, frequently lacking detailed descriptions of operative technique and exhibiting substantial heterogeneity in outcome reporting. Such variability precludes meaningful meta-analysis and impedes the development of evidence-based consensus guidelines. The prevailing “binary” approach seen in the literature towards robotic adoption, wherein surgeons either utilise the robot in all or none of their cases, highlights the urgent need for a more nuanced, phenotype-based surgical strategy.

A recurring theme was the disproportionate focus on technically “simpler” IBD cohorts—particularly patients with normal-range body mass indices and limited disease complexity [26]. Whilst this finding is likely attributable to the ongoing learning curve and adoption of IBD surgeons to the robotic surgery platform, the reported case preference also introduces selection bias and limits the generalisability of reported outcomes to the broader IBD population. Clinically, this cohort of patients more typically includes individuals with prior surgical interventions, malnutrition, steroid use, and complex or penetrating disease phenotypes. Furthermore, follow-up intervals were frequently truncated, thereby weakening the interpretability of long-term outcomes such as reoperation rates and disease recurrence—critical endpoints given the chronic, relapsing nature of IBD.

Geographic bias also emerged as a salient issue, with the majority of studies originating from high-income countries, particularly the United States. This reflects disparities in access to robotic surgical platforms, which remain concentrated in well-resourced, high-volume tertiary centres [27]. In contrast, low- and middle-income settings face substantial barriers to implementation due to the capital-intensive nature of robotic infrastructure. These systemic inequities—rarely addressed in the current literature—raise concerns about the broader applicability and equity of robotic surgical innovation.

There was also substantial variability in reported intraoperative strategies across studies, encompassing differences in port placement, docking preferences, anastomotic configurations, and approaches to bowel mobilisation. However, a consistent limitation throughout the literature is the poor granularity in technical descriptions—particularly concerning access strategies, trocar positioning, and robotic system docking. These omissions are especially problematic given that robotic surgery in IBD remains a relatively new and evolving technique. Access and setup steps are critical determinants of procedural success, and in this context, where disease distribution and prior operations often necessitate nuanced and flexible configurations; limited detail in reporting of these domains significantly limits reproducibility. It also inhibits meaningful comparison across centres and ultimately hinders the development of training and standardised approaches.

Robotic surgery facilitates precise pelvic dissection within confined anatomical regions, particularly in completion proctectomies or restorative procedures for UC, even with a hybrid laparoscopic/robotic approach [28]. However, its utility may be more limited for subtotal colectomy, where multi-quadrant dissection and repeated redocking are often necessary. The robotic platform’s enhanced visualisation and increased instrument articulation are well suited to facilitate nerve preservation, improve accuracy of mesorectal dissection, and support secure anastomosis formation. In contrast to the anatomical variability and operative complexity often encountered in Crohn’s disease, surgery for ulcerative colitis is typically more standardised, localised, and anatomically predictable. This may support the development of reproducible robotic workflows and makes anatomically confined procedures a suitable target for the establishment of consensus guidelines and procedural benchmarks. Further prospective studies focusing on technical standardisation and functional outcomes are warranted to better define these potential advantages and optimise implementation.

Where reported, the majority of included studies utilised fourth-generation da Vinci platforms (da Vinci Xi and X), which represent the current workhorses of contemporary robotic colorectal surgery. Operative site is also an important determinant of robotic case selection. Historically, robotic training has largely been industry-led and structured around a stepwise learning curve in which left-sided and single-field pelvic procedures are prioritised earlier, with more complex right-sided, multi-quadrant, and extended colectomies undertaken later. As a result, these procedures may be less likely to be attempted robotically and less commonly published leading to potential under-representation in the published literature.

Furthermore, definitions of postoperative outcomes remain inconsistent or are omitted altogether, with key clinical endpoints (e.g., ‘pouch failure’) often subjectively defined and unstandardised. This lack of uniformity impedes accurate benchmarking and synthesis of results across studies. Several studies highlighted the potential advantages of robotic platforms in technically complex scenarios. For instance, Calini et al. demonstrated comparable rates of ileus, length of stay, and overall complications between patients undergoing intracorporeal and extracorporeal anastomoses, despite the extracorporeal cohort being significantly more comorbid (higher rates of American Society of Anaesthesiologists Score 3) [11]. These benefits are especially relevant in CD, where dense adhesions, mesenteric foreshortening, and inflammation complicate laparoscopic dissection. Notably, conversion rates from robotic to open surgery were substantially higher in patients with complex or recurrent CD, reaching up to 37% in some series, compared to approximately 14% in less complicated cases [29]. Despite this, studies in this review studies suggest that robotic reoperations in IBD can be performed with safety and efficacy comparable to primary procedures. These favourable outcomes are largely attributed to individualised operative planning—particularly preoperative imaging to guide trocar placement and the use of dual docking strategies to facilitate multi-quadrant access [14, 24]. 

Preoperative optimisation remains underemphasised in the current literature. IBD patients—particularly those with CD—frequently present in catabolic states with significant nutritional deficiencies, which are risk factors for poor postoperative outcomes. Current European Crohn’s and Colitis Organisation (ECCO) guidelines recommend multidisciplinary preoperative optimisation, including nutritional support and inflammatory control, yet few studies included in this review reported detailed perioperative care protocols [30]. This is particularly important given the well-established relationship between thorough preoperative enteral nutrition leading to reduced postoperative morbidity [31]. 

Importantly, adherence to ERAS protocols has been associated with reduced rates of anastomotic leak, postoperative sepsis, and shorter hospital stays—even in reoperative IBD cases [13, 14]. These results are strengthened by rigorous study designs, including the use of validated classification systems such as Montreal and Clavien-Dindo classifications and the exclusion of confounding factors [32, 33]. Despite these clear benefits, ERAS compliance was inconsistently reported and suboptimally implemented across the included studies. Given the well-documented positive impact of ERAS on perioperative outcomes in colorectal surgery more broadly [34–37], greater emphasis on ERAS in this specific context is needed in the literature. Wider adoption and standardised reporting of ERAS components are warranted to improve postoperative recovery in this high-risk cohort.

While robotic surgery appears to offer modest short-term advantages—such as reduced conversion rates and expedited functional recovery—definitive evidence of clinical superiority over laparoscopy remains debated [37]. Moreover, these benefits may be offset by significantly higher procedural costs, largely driven by increased operative time and capital expenses [38]. However, current cost-effectiveness analyses are limited in scope, often focusing solely on intraoperative or immediate postoperative metrics. There is a need for comprehensive economic evaluations that incorporate long-term outcomes such as disease recurrence, reoperation rates, quality-adjusted life years (QALYs), and return to work—particularly relevant in CD, where patients often face a high lifetime surgical burden.

A notable gap in the literature is the absence of validated patient-reported outcome measures (PROMs). Given the chronic, quality-of-life-altering nature of CD and the supportive, rather than curative, role of surgery, PROMs are essential to evaluating the real-world impact of surgical intervention. The inclusion of standardised PROMs assessing psychological well-being, functional capacity, and overall satisfaction would enhance the patient-centredness of future research.

Future research should prioritise the establishment of multicentre, prospective registries, capturing granular intraoperative data and perioperative variables across high-risk subgroups—including patients with prior operations, fistulising disease, or pelvic sepsis. Further adherence to rigorous reporting standards that incorporate detailed intraoperative methodologies, including port mapping and docking strategies, alongside consistent outcome definitions, extended follow-up, and adjustment for key confounders are needed. Coupled with PROMs and long-term follow-up, such datasets would meaningfully inform both technical refinement and policy development in robotic IBD surgery.

## Conclusions

The robotic surgical literature in IBD remains limited by methodological heterogeneity, geographic and selection biases, and inconsistent outcome reporting. The lack of procedural standardisation and underutilisation of patient-centred and long-term endpoints significantly constrains the ability to draw generalisable, clinically meaningful conclusions. Addressing these deficiencies through prospective, multicentre studies with standardised protocols, validated PROMs, and long-term follow-up is critical to fully characterising the role of robotic surgery in the management of CD.

## Figures and Tables

**Fig. 2:**
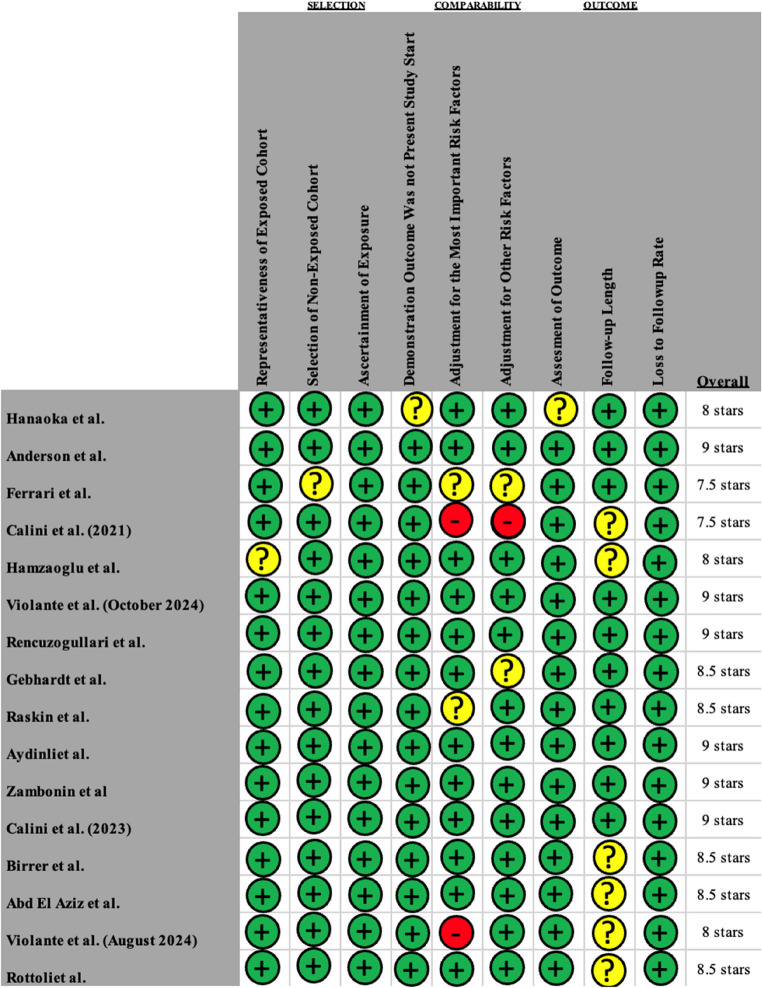
Risk of bias assessment of included studies using a modified Newcastle-Ottawa Scale. This figure presents the risk of bias assessment for each included observational study using a modified version of the Newcastle-Ottawa Scale (NOS). Risk domains were evaluated and colour-coded as follows: green (+) indicates low risk of bias, red (-) indicates high risk of bias, and yellow (?) indicates unclear risk. A scoring system was applied, with a penalty of 1 point for each high-risk domain (red) and 0.5 point for each unclear domain (yellow). No penalty was applied for low-risk assessments (green)

## Data Availability

No datasets were generated or analysed during the current study.

## Appendix

Newcastle-Ottawa Scale (NOS) Quality Assessment:

See Fig. 2.
